# Platelet responses to agonists in a cohort of highly characterised platelet donors are consistent over time

**DOI:** 10.1111/vox.12468

**Published:** 2016-12-21

**Authors:** S. F. Garner, A. Furnell, B. C. Kahan, C. I. Jones, A. Attwood, P. Harrison, A. M. Kelly, A. H. Goodall, R. Cardigan, W. H. Ouwehand

**Affiliations:** ^1^ NHS Blood and TransplantCambridgeUK; ^2^ Department of HaematologyUniversity of CambridgeCambridgeUK; ^3^ Pragmatic Clinical Trials UnitQueen Mary University of LondonLondonUK; ^4^ Institute for Cardiovascular and Metabolic ResearchUniversity of ReadingReadingUK; ^5^ Institute of Inflammation and AgeingQueen Elizabeth HospitalUniversity of BirminghamBirminghamUK; ^6^ Department of Cardiovascular SciencesUniversity of LeicesterLeicesterUK; ^7^ NIHR Cardiovascular Biomedical Research UnitGlenfield HospitalLeicesterUK; ^8^ Wellcome Trust Sanger InstituteCambridgeUK

**Keywords:** donors, platelet components, platelet function

## Abstract

**Background and Objectives:**

Platelet function shows significant inheritance that is at least partially genetically controlled. There is also evidence that the platelet response is stable over time, but there are few studies that have assessed consistency of platelet function over months and years. We aimed to measure platelet function in platelet donors over time in individuals selected from a cohort of 956 donors whose platelet function had been previously characterised.

**Materials and Methods:**

Platelet function was assessed by flow cytometry, measuring fibrinogen binding and P‐selectin expression after stimulation with either cross‐linked collagen‐related peptide or adenosine 5′‐diphosphate. Eighty‐nine donors from the Cambridge Platelet Function Cohort whose platelet responses were initially within the lower or upper decile of reactivity were retested between 4 months and five and a half years later.

**Results:**

There was moderate‐to‐high correlation between the initial and repeat platelet function results for all assays (*P* ≤ 0·007, *r* ^2^ 0·2961–0·7625); furthermore, the range of results observed in the initial low and high responder groups remained significantly different at the time of the second test (*P* ≤ 0·0005).

**Conclusion:**

Platelet function remains consistent over time. This implies that this potential influence on quality of donated platelet concentrates will remain essentially constant for a given donor.

## Introduction

Within the general population, several studies have confirmed the long‐held notion that the response of platelets to agonists is highly variable, but for an individual, the level of responsiveness is remarkably consistent over time 1, 2, 3, 4, 5. The belief that highly characterised platelet function phenotypes are stable has underpinned the application of genomic studies to identify genetic variants underlying platelet function 6, 7, 8.

The recognition that platelet reactivity is at least partly genetically controlled, and therefore, potentially stable over time is supported by observations in the Framingham Heart Study which found that heritable factors played a key role in aggregation responses 9 and is compatible with the notion that the interindividual variation in platelet parameters such as their count, volume and function is to a large extent inherited and therefore stable 7, 8, 10, 11, 12, 13.

Knowledge that platelet function is consistent over time has a number of implications. The ability to reproducibly measure and demonstrate stable platelet function for a given individual supports the desire to personalise treatment with antiplatelet therapy after percutaneous coronary intervention 14. Similarly, demonstration of wide but stable variation of platelet function between individuals has specific implications for blood services, as inherent variation in donor platelet function has already been shown to influence the quality of platelets donated via apheresis 15. Furthermore, donor variability has also been suggested as a factor that influences posttransfusion platelet increments 16.

Many studies of platelet function however only cover periods of months, and some focus on single agonists, single measures of activation or only on individuals defined as hyper‐responsive to specific agonists 1, 2, 5. Similarly, although variation in platelet function among blood and particularly platelet donors has been demonstrated 3, 17, there is a lack of information about the consistency of this variation over time. Our previous study of both whole blood and plateletpheresis donors demonstrated wide but consistent interindividual variation in platelet function. It however only assessed reproducibility after 3 months and did not review the details of blood donations between the initial and repeat testing 3.

The overall aim of our study was to measure platelet function in a cohort of individuals over a period of time which was longer than previously reported, to support the notion that platelet function within an individual is consistent over time. We achieved this with a panel of 89 established platelet donors whose platelet function was known to be at either the low or high ends of the normal range and testing them at intervals between measurements ranging from 4 months to approximately 5 years. If observed, demonstration of consistent platelet function over these periods would add to existing knowledge and achieve our aims of providing further support to the belief that this trait is at least partially genetically controlled and consequently indicating that any association between a donor's platelet function and the quality of their donated platelets would remain a consistent feature. In addition, the data would support the use of donors with highly characterised platelet function in clinical trials to assess the role of donor variation on the outcome of transfusions.

## Materials and methods

### Cohorts and subjects

The subjects for this study were selected from the 956 whole blood or platelet donors in the Cambridge Platelet Function Cohort 3. They were recruited over a period of 5 years, from the National Health Service Blood and Transplant blood donor clinic in Cambridge after gaining informed, written consent, and were established donors of platelets by apheresis. The study was approved by the Huntingdon Research Ethics Committee (Reference number 05/Q0104/27*)*.

Individuals in the entire cohort of 956 donors were characterised on the basis of their platelet response to cross‐linked collagen‐related peptide (CRP‐XL) and ADP, assessing their activation by flow cytometry, with fibrinogen binding and P‐selectin (CD62P) expression as markers representing activation of the glycoprotein IIbIIIa complex and α‐granule release, respectively. These tests were carried out in the presence of aspirin and hirudin, to block the effects on the platelets of endogenous thromboxane A2 and thrombin, respectively, and in the case of the CRP‐XL‐stimulated samples, apyrase was added to block endogenous ADP.

Initial testing of the donors occurred during two periods. Platelets from donors in the first period, from July to August 2005, were tested against both agonists using both activation markers giving four measurements of platelet function. The second recruitment period, between June 2009 and July 2010, occurred in order to increase the size of the cohort to provide platelets for use in a clinical trial investigating possible associations between the level of platelet responsiveness and the clinical efficacy of transfused platelets (http://www.nhsbt.nhs.uk/clinicaltrialsunit/current-trials/prompt/index.asp#.Vl7Ot8J_t1E). During this period, fibrinogen binding in response to both ADP and CRP‐XL was measured, but P‐selectin expression was only measured following CRP‐XL activation, giving three platelet function measurements. Completion of the second recruitment period 5 years after the initial recruitment began provided the opportunity to retest donors with a range of different time periods between their initial and repeat testing. The assays used for the original testing were repeated to assess the consistency of platelet function over time.

**Figure 1:**
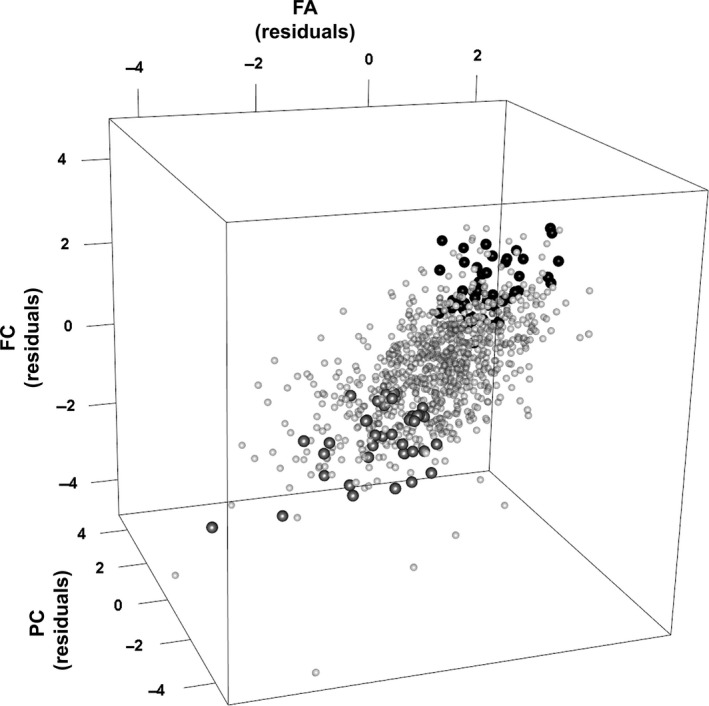
Platelet function responder groups. Values represent the standardised residuals of the logit‐transformed results for fibrinogen binding in response to ADP stimulation (FA), fibrinogen binding in response to CRP‐XL stimulation (FC) and P‐selectin expression following CRP‐XL stimulation (PC) for the entire cohort of 956 donors. The cohort excluding the selected low and high responders is shown as light‐grey spheres. Donors classified as low responders at first testing and still available for repeat testing as shown as mid‐grey spheres and the available high responders as black spheres.

The 89 platelet donors identified for retesting were selected for the clinical trial based on the hypothesis that use of donors with either low or high platelet function would be most likely to demonstrate potential differences in clinical outcome in patients. These donors had been characterised as having platelet reactivity within the lower or upper decile of overall observed reactivity. The method for assigning the donors to these low and high responder groups has been previously described 3. Briefly, classification involved combining the standardised residuals of the logit‐transformed flow cytometry data for all measurements into a single ranking profile. High responders were defined as those with the highest minimum response, low responders as those with the lowest maximum. The donors were therefore considered as having overall platelet function responses that were either low or high, based on the overall range of responses observed in the entire cohort. For illustration, Fig. 1 shows where the selected donors lie in relation to the whole cohort in the distribution of results for the three assays common to testing all donors.

Of the 89 donors, 66 (28 low responders and 38 high responders) who had been initially tested between 4 and 20 months earlier had donated an average of eight times during this period. The remaining 23 (nine low responders and 14 high responders) were from the first recruitment period, originally tested between 64 and 68 months earlier and were described in 2007 3. These individuals donated an average of 49 times between initial and repeat testing. Characteristics of the 89 donors are shown in Table 1. All the donors were Caucasians, and none had diabetes.

**Table 1:** Details of the short‐term and long‐term follow‐up groups

Characteristic	Short‐term	Long‐term
Number of donors	66	23
Mean number of donations between tests (range)	8 (1–22)	49 (4–80)
Number of low responders (%)	28 (42)	9 (39)
Number of high responders (%)	38 (58)	14 (61)
Mean age, years at initial test (SD)	49·3 (9·4)	47 (6·7)
Mean age, years at repeat test (SD)	50·3 (9·4)	52 (6·7)
Sex, female: male	4:62	1:22
Mean body mass index (SD)	27·4 (4·1)	28·0 (3·5)
Current smoker (%)	6 (9)	1 (4)
Subjects with hypertension (%)	4 (6)	0
Subjects with high cholesterol (%)	5 (8)	4 (17)
Contraceptive pill (%)	1 (2)	0

### Blood collection

Blood was drawn prior to apheresis donation, from the antecubital fossa contra lateral to the one used for routine donation. A 21‐gauge butterfly needle and a Vacuette tube (Griener Bio one, Stonehouse, UK) were used following a standardised protocol. The first 3 ml of blood were discarded, and a subsequent sample was taken into 3·2% sodium citrate for platelet function analysis.

### Platelet activation reagents

For platelet activation, CRP‐XL (monomeric sequence GCO[GPO]_10_GCOG) was prepared as described previously 18 and was a kind gift from Professor Richard Farndale. Different batches of CRP‐XL were used for the initial and repeat testing. Due to the instability of the diluted peptide, the reagent was stored in concentrated form at +4°C and diluted to a working concentration each day. The same batch of ADP, obtained from Sigma Chemical Co., Ltd (Poole, Dorset, UK), was used throughout the study and stored at −80°C in single‐use aliquots.

The inhibitors, aspirin, apyrase and hirudin, were all from Sigma and were stored at −80°C in single‐use aliquots until added to the citrated blood during the activation assays.

The antibodies used for flow cytometry were rabbit polyclonal FITC‐anti‐fibrinogen (Dako Ltd, Ely, UK) and a single batch of PE‐anti‐CD62P (Bristol Institute for Transfusion Science, Bristol, UK). HEPES‐buffered saline (HBS, 0·14 m NaCl, 5 mm KCl, 1 mm MgSO_4_, 10 mm HEPES (sodium salt) pH 7·4) was used for all dilutions.

### Platelet activation measurements using whole blood flow cytometry

Blood was processed within 10 minutes of venesection. Aspirin (100 μm) and hirudin (10 U/ml) were added to an aliquot of citrated blood and 5 μl of this added to 45 μl of HBS containing CRP‐XL (0·1–0·5 μg/ml, depending on batch) or ADP (10^−7^ m) and either FITC‐anti‐fibrinogen, or PE‐anti‐CD62P. For CRP‐XL‐stimulated samples, apyrase (4 U/ml) was also included in the reaction mixture. Samples were incubated for 20 minutes at room temperature, and reactions were then stopped by 100‐fold dilution in formyl saline (0·2% formaldehyde in 0·9% NaCl). Negative controls for the P‐selectin antibody were set using an appropriate isotype control and for antifibrinogen using samples incubated with the antibody in the presence of 10 mm EDTA. Flow cytometric analysis was carried out on either an EPICS Profile XL (Beckman‐Coulter Ltd., High Wycombe, UK) for the initial testing period or a FC500 flow cytometer (Beckman‐Coulter Ltd) for the second testing period. Platelets were identified by light scatter, and results were recorded as the percentage of platelets positive for the relevant activation marker. All tests were performed in duplicate, and the average result were used for analysis.

The protocol was as referred to previously 3, with the exceptions that the final reaction volume was changed to 50 μl, and the final concentration of EDTA to 10 mm.

### Statistical analysis

Data were analysed using prism versions 4.0c (GraphPad Software Inc, San Diego, USA). The correlation between initial and repeat platelet function results was analysed using Pearson's correlation, and differences between the responder groups were compared using an unpaired *t*‐test; *P* values less than 0·05 were considered statistically significant.

## Results

### Platelet function results

**Figure 2:**
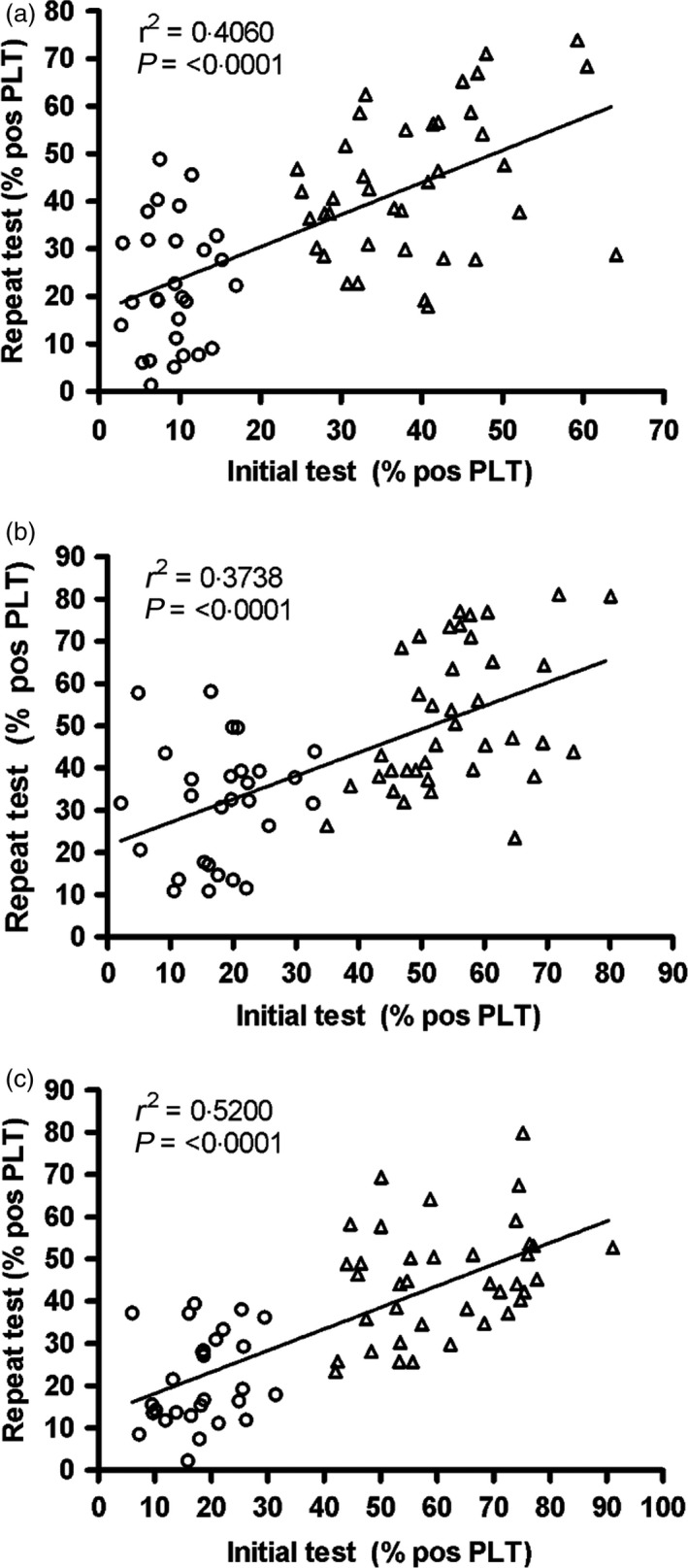
Short‐term follow‐up (4–20 months): relationship between initial and repeat platelet function test results. Results for the first and repeat tests are shown. Values represent the percentage of platelets (PLT) expressing the relevant activation marker. Donors classified as low responders at first testing are shown as ○ and high responders as ▵. (a) Fibrinogen binding following CRP‐XL stimulation, (b) P‐selectin expression following CRP‐XL stimulation and (c) fibrinogen binding in response to ADP stimulation (*n* = 66 donors for all tests).

Platelets from both the low and high responder donors who were followed up over a short term (4–20 months) were analysed for fibrinogen binding following stimulation with ADP and CRP‐XL and for P‐selectin expression following CRP‐XL induced activation. As shown in Fig. 2, the level of reproducibility of the three assays was moderate, with significant correlations being observed between the original and retest data for all assays (*r* ^2^ > 0·37; *P* < 0·0001 for all).

**Figure 3:**
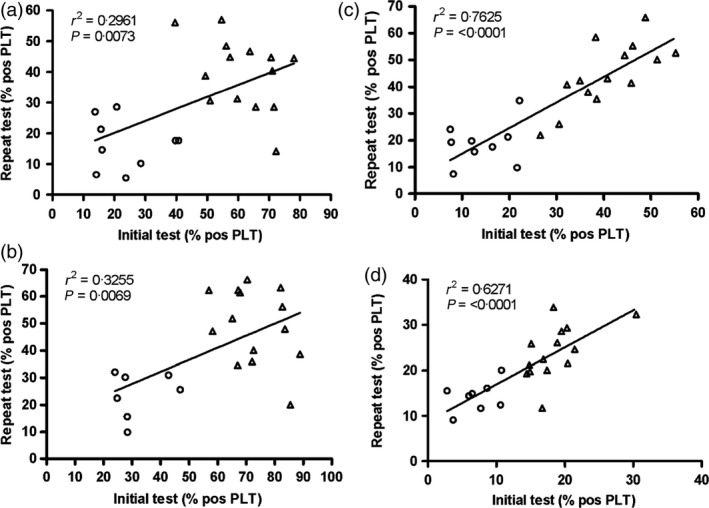
Long‐term follow‐up (64–68 months): relationship between initial and repeat platelet function test results. Results for the first and repeat test are shown. Values represent the percentage of platelets (PLT) expressing the relevant activation marker. Donors classified as low responders at first testing are shown as ○ and high responders as ▵. (a) Fibrinogen binding following CRP‐XL stimulation (*n* = 23 donors), (b) P‐selectin expression following CRP‐XL stimulation (*n* = 21 donors), (c) fibrinogen binding in response to ADP stimulation (*n* = 23 donors) and (d) P‐selectin expression in response to ADP stimulation (*n* = 22 donors).

Donors who were followed up for a longer term (64–68 months) were analysed for both fibrinogen binding and P‐selectin expression measured following stimulation with CRP‐XL and ADP. Again, significant correlations were observed between the original and retest data for all four assays (*r* ^2^ > 0·29; *P* ≤ 0·0073, Fig. 3).

For both groups, it was notable that the ADP results were more reproducible than those obtained with CRP‐XL, with higher degrees of correlation and lower *P*‐values (Figs 2 and 3).

### Maintenance of low and high responder status

Despite the high degree of correlation between the initial and retest results, there were some divergence and regression to the mean. Therefore, we tested whether the classification of groups as low and high responders remained valid over time.

At the initial testing, results from all assays were significantly different between the two responder groups. On retesting the donors with a shorter‐term follow‐up, between 4 and 20 months later, the differences between responder groups for the three tests performed remained significant. Similarly, for the long‐term donors, retesting more than 5 years later again revealed significant differences between the two responder groups for all four tests at both time‐points (Table 2).

**Table 2:** Result ranges for low and high responder donors^a^

*Group*	Short term (*n* = 66)				Long term (*n* = 23)			
Test occasion	Initial		Repeat		Initial		Repeat	
Responder group	Low	High	Low	High	Low	High	Low	High
Agonist (activation marker)								
CRP‐XL (Fibrinogen)	9·1 ± 3·6	38·9 ± 10·1	22·2 ± 13·2	43·9 ± 15·3	23·7 ± 10·6	61·5 ± 10·8	16·6 ± 8·2	39·6 ± 11·8
CRP‐XL (P‐selectin)	17·9 ± 7·7	55·5 ± 9·9	31·4 ± 14·1	52·2 ± 16·7	31·8 ± 9·1	72·9 ± 10·1	26·8 ± 11·0	49·1 ± 13·8^b^
ADP (Fibrinogen)	18·2 ± 6·6	61·5 ± 12·8	21·2 ± 10·8	45·1 ± 13·1	14·2 ± 5·9	40·8 ± 8·3	18·9 ± 8·0	44·5 ± 12·1
ADP (P‐selectin)	nt	nt	nt	nt	7·1 ± 2·9	18·6 ± 4·1	13·8 ± 3·5	24·0 ± 5·8

nt, not tested.

^a^Results are the percentage of platelets expressing the relevant activation marker, and values are mean ± 1SD.

The difference between low and high groups was all significant with *P* < 0·0001, except for^b^ where *P* = 0·0005.

## Discussion

To date, studies of variation in platelet function that have included monitoring function over time have demonstrated consistency over periods ranging mainly from weeks to months, with a small number being confirmed after an average of 19 months 1, 2, 3, 5. These observations are in keeping with the notion that this quantitative trait is strongly genetically controlled.

We have added to the data supporting this assumption by expanding our previously described Cambridge Platelet Function Cohort 3 to include almost one thousand donors and subsequently retesting a subset of individuals sampled from the tail ends of the normal distribution of platelet function, and which were considered as either low or high responders.

By assessing volunteers twice, with test intervals between 4 and 68 months and after making between one and 80 donations, we were able to demonstrate consistency of platelet functional responses within individuals. Furthermore, the significant differences in platelet function between low and high responders at the time of initial enrolment were maintained. These observations imply that regular platelet donation does not have a major long‐term impact on platelet function in established donors. They do not however exclude temporary effects immediately following donation. For example, we have previously shown that platelets remaining in the donor's circulation after apheresis donation were generally less responsive to ADP than before donation 15.

Although the initial and second assay results correlated significantly, they were however not identical. A tendency for regression to the mean, where results which are low or high at first measurement tend to be closer to the average result on second measurement, may have contributed to differences in results. In addition, technical and operational variables between the two occasions of testing could have influenced results. The greater reproducibility of the ADP results compared with the CRP‐XL results for example probably reflects batch variability for CRP‐XL, whereas the same batch of ADP was used for all tests.

Furthermore, although it is believed that up to 70% of variation in platelet responses is inherited 8, 9, 19, circadian variations, environmental factors such as diet, smoking and exercise can also contribute to small variations platelet function 20. These factors were not controlled within the current study. The ability to improve the reproducibility of platelet function assay results by regulating factors such as food, alcohol intake and exercise was highlighted by previous observations that within an individual, variability in platelet function could be decreased by standardising these parameters 21.

Demonstration of wide but stable variation of platelet function may have implications for transfusion therapy. Previous studies have shown the degree of activation induced by collecting platelets via apheresis procedures varies considerably between donors 22, 23. We have demonstrated that the inherent platelet responsiveness of individual donors may contribute to platelet quality, with highly activated platelets being obtained from high responder donors 15. Thus, variation in quality of platelet concentrates is at least partly donor related, and as such our current study implies that this will remain a consistent feature for a given donor.

The clinical implications of the current observations for patients receiving platelet transfusions however remain unclear. There is still debate about the ability of *in vitro* platelet function measurement to predict *in vivo* platelet recovery, survival and function. Some reports have suggested that expression of activation markers such as CD62P in platelet concentrates correlates inversely with posttransfusion survival 24, while others have failed to find such a relationship 25. Data from the recently completed semi‐randomised, controlled clinical trial to assess whether the outcome from platelet transfusions differs according to the platelet function characteristics of the donor are currently being reviewed (http://www.nhsbt.nhs.uk/clinicaltrialsunit/current-trials/prompt/index.asp#.Vl7Ot8J_t1E).

Overall, we have shown that the wide variation of platelet function among blood donors remains consistent over periods of up to 68 months of regular donations. This observation is compatible with the notion of there being a high level of heritability for this quantitative platelet trait, and this may have implications for platelet transfusions, as well as wider considerations in relation to haemostasis and thrombosis.

## Author contributions

Stephen F. Garner assisted in the study design, performed laboratory assays, analysed data and wrote the paper. Abigail Furnell performed laboratory assays and analysed data. Brennan. C. Kahan contributed to the study design and performed statistical analysis. Chris I. Jones assisted in the study design, performed laboratory assays, analysed data and cowrote the paper. Antony Attwood maintained laboratory and donor databases and analysed donor data. Paul Harrison assisted in the study design, reviewed laboratory data and cowrote the paper. Anne M. Kelly performed laboratory assays, reviewed data and recruited and consented volunteers. Alison H. Goodall, assisted in the study design, analysed data and cowrote the manuscript. Rebecca Cardigan assisted in the study design, analysed data and cowrote the manuscript. Willem H. Ouwehand, designed and supervised the study, and cowrote the manuscript.

## Conflict of interests

The authors declare no Conflict of interests.
